# Determination of behavior of catalpol hexapropionate in simulated gastric conditions by UPLC–ESI–HRMS

**DOI:** 10.1038/s41598-020-68056-5

**Published:** 2020-07-07

**Authors:** Xiaodong Cheng, Qiuxia Zhang, Zhenxing Li, Chunhong Dong, Shiqing Jiang, Yu-an Sun, Guoqing Wang

**Affiliations:** 1 School of Materials and Chemical Engineering, Zhengzhou University of Light Industry, Zhengzhou, China; 2 School of Chemical and Environmental Engineering, Pingdingshan University, Pingdingshan, China; 3 Henan University of Chinese Medicine, Zhengzhou, China

**Keywords:** Chemistry, Analytical chemistry, Medicinal chemistry

## Abstract

Catalpol hexapropionate (CP-6) was designed and synthesized as anti-aging drug. In order to investigate the behavior of CP-6 in simulated gastric juice, ultra-high performance liquid chromatography–electrospray ionization–high resolution mass spectrometry was used to determinate the components produced in simulated gastric conditions. Six metabolites were identified with the possible metabolic processes proposed. Hydrolysis may be the main metabolic pathways. The relative contents of CP-6 and its metabolites were determined using their extractive ion chromatograms. The results show that the relative content of CP-6 is rapidly decreased about 15% during the first 0.5 h and generally stable after 0.5 h. The mainly produced metabolites are catalpol penta-propionate (CP-5), catalpol and a spot of catalpol tetra-propionate (CP-4), catalpol tri-propionate (CP-3), catalpol dipropionate (CP-2) and catalpol propionate (CP-1). The metabolitic process of CP-6 may be an hydrolysis under acid conditions. The research results can provide useful information for development and utilization of CP-6 as a pharmaceutical preparation.

## Introduction

Catalpol is a small molecular of iridoid glycoside which can be derived from fresh or dried root of the *rehmannia glutinosa Libosch*^1^. In recent years, catalpol has been paid more and more attention because of its extensive pharmacological activities. A large number of studies have shown that catalpol has a good pharmacological activity in improving cardiovascular, cerebrovascular, central-nervous system diseases and boosting immunity, regulating blood glucose and lipid metabolism, anti-tumor, anti-osteoporosis, anti-inflammation^2–13^. However, the application of catalpol was severely limited in clinic because of its short half-life in vivo and high water solubility, it is difficult to penetrate the blood–brain barrier^14^In order to improve its druggability, the structure of catalpol can be modified by esterification, and we previously introduced propionic anhydride to obtain catalpol propionylated derivatives, and the molecular docking study (MD) and MTT analysis showed that the catalpol hexapropionylation derivative (CP-6) maybe with the highest anti-aging effect^15,16^.

It is known that the metabolism of oral drugs in the gastrointestinal tract will directly affect the amount of maternal drugs in the digestive tract, in which drugs may undergo chemical degradation or enzyme degradation, thus reducing the amount of maternal drugs and reducing their bioavailability^17,18^. The methods of drug metabolism research include two methods: in vivo research and in vitro research. Because of the simplicity and rapidity of in vitro metabolism research, it was more and more used to detect the complex physical and chemical changes of drugs in the digestive tract, especially the conjecture of their metabolic pathways and the identification of metabolites. The conventional methods for in vitro metabolic studies include high performance liquid chromatography (HPLC–UV), micellar electric capillary chromatography-mass spectrometry (MECC-MS), gas chromatography-mass spectrometry (GC–MS), GC–MS/MS and liquid chromatography-mass spectrometry (LC–MS) or LC–MS/MS^19–24^. In these methods, the metabolites should be previously separated by GC or LC, and then be identified by UV, MS or MS/MS methods. These preseparated or MS/MS identified procedures are usually time consuming.

In this work, using UPLC as means of separation and sampling device, ESI as ionizing device characteristic with no gas ionized debris, and Orbitrap-Exactive high resolution mass spectrometer (HRMS) for accurate determination of molecular mass of organic compounds^25^, the metabolic process of CP-6 in simulated gastric conditions was determined, and the determined metabolites may provide useful information for the development and utilization of CP-6 as pharmaceutical preparation.

## Experimental

### Materials and regents

Catalpol hexapropylate was laboratory homemade and purified (CP 98%). Chromatographic pure reagent MeOH, CH_3_CN and ethyl acetate were purchased from Tianjin Siyou Fine Chemicals Co. Ltd., China. Ultrapure water was prepared by Mill-QAdvantage A10 ultrapure water meter (Millipore, USA). Pepsin (Saiguo Biotechnology Co. Ltd., China, 1:10,000). Trypsin (Jiangsu Kaiji Biotechnology Co. Ltd., China, 1:250). HCl (Nanjing Chemical Reagent Co. Ltd., China, AR 36.0%-38.0%). NaOH (Xilong Chemical Co. Ltd., China, AR 99%). KH_2_PO_4_ (Nanjing Chemical Reagent 1st Plant, China, AR 99%).

Standard stock solution of CP-6 (2 mg/mL) was prepared using 200 mg CP-6 and MeOH to dissolve with the final volume completed to 100 mL.

Simulated gastric juice was prepared using 0.70 mL HCl, 0.20 g NaCl and 0.32 g pepsin which dissolved in distilled water, and the final solution is completed constant volume to 100 mL with distilled water. The resulting solution with a pH value of ~ 1.2 was maintained at 37 °C and stirred continuously^21,26^.

### Apparatus

Waters ACQUITY Ultra High Performance Liquid Chromatograph coupled with Thermo Fisher-Exactive Obitrap high resolution mass spectrometer (HRMS) was used for simulated gastric juice and simulated intestinal fluid analysis. DF-101 SA-H collecting heating at constant temperature magnetic stirrer (Nanjing Kohl Instruments and Equipment Co. Ltd., China), 80–2 Electric Centrifuge (Changzhou Guoyu Instruments Manufacturing Co. Ltd., China).

UPLC conditions: Waters ACQUITY UPLC with Wakopak ultra C18-3 (3.0 mm × 250 mm) column and PDA detector.

HRMS conditions: ESI ionization source; automatic sampler mobile phase ratio, acetonitrile:0.1% aqueous formate solution (V:V) = 60:40; flow rate, 200 μL/min; capillary temperature, 250 °C; capillary tube voltage, 60 V; tube voltage, 120 V; injection volume, 0.1 μL; auxiliary gas, 10 L/min; sheath gas, 40L/min; skimmer voltage, 22 V; sketch time, 2 min; scan range/(m/z), 100–1,000, electronic transmission tube temperature, 275 °C; scanning mode, positive and negative ions scanning mode respectively.

### Determination of the metabolites

2 mL stock solution of CP-6 prepared in section *Materials and Regent* was added to 5 mL simulated gastric juice which were placed at 37 °C thermostatic bath. Respectively sampling 200 μL at 0, 0.05, 0.5, 1.0, 1.5, 2.0, 2.5, 3.0, 3.5 and 4.0 h, adding 0.2 mol/L NaOH solution to adjust pH ~ 7, adding 1 mL MeOH and mixing to deactivate the enzyme^21,26^. Centrifugated 1,000 r/min for 15 min, filtered with 0.22 μm PTFE microporous membrane, then 0.1 μL filtered solution was analyzed using the optimal UPLC-ESI-HRMS conditions.

## Results and discussion

### Selection of ion scanning mode

**Figure 1:**
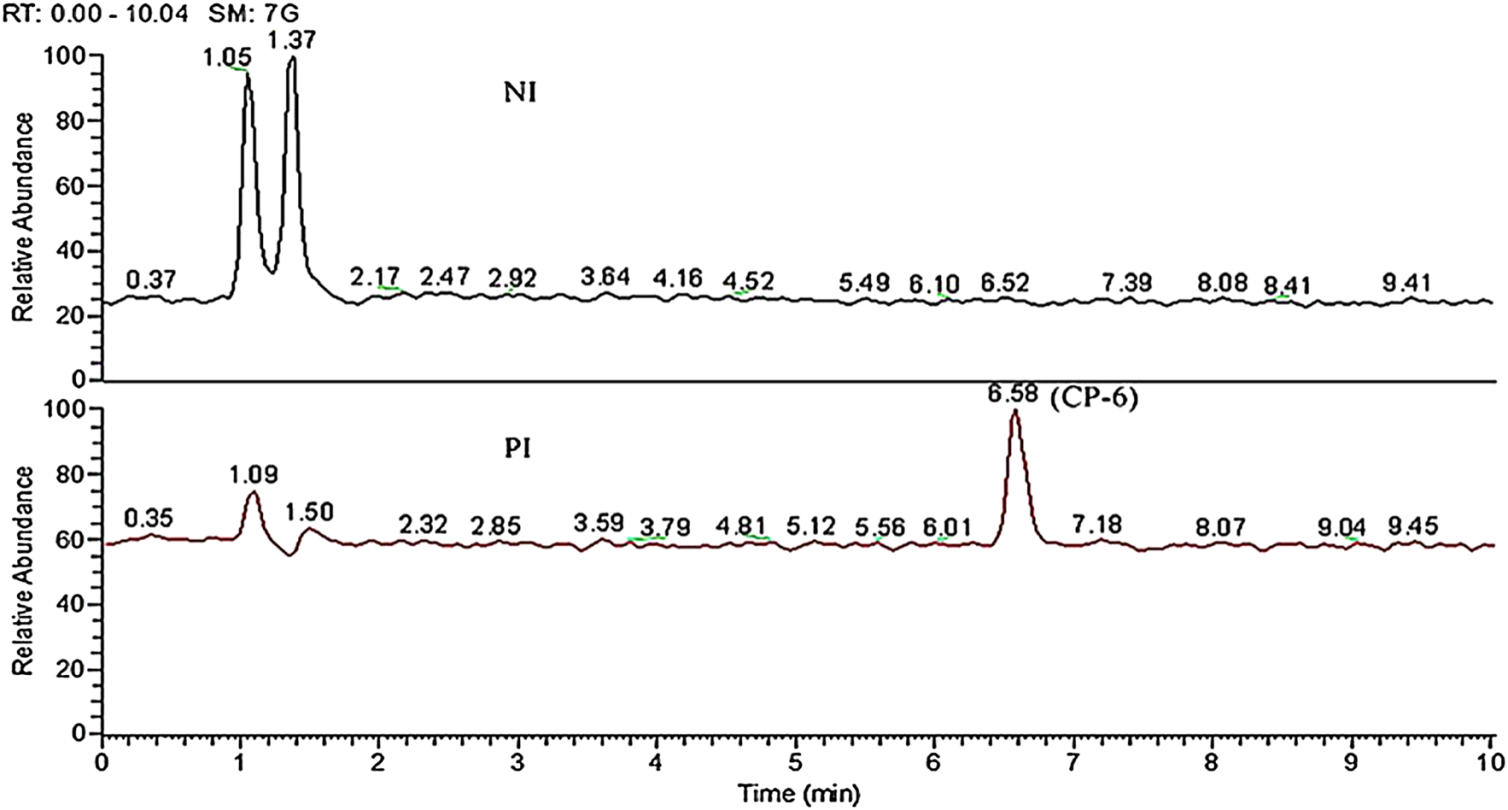
TIC of metabolites analyzed at NI mode and PI mode.

**Figure 2:**
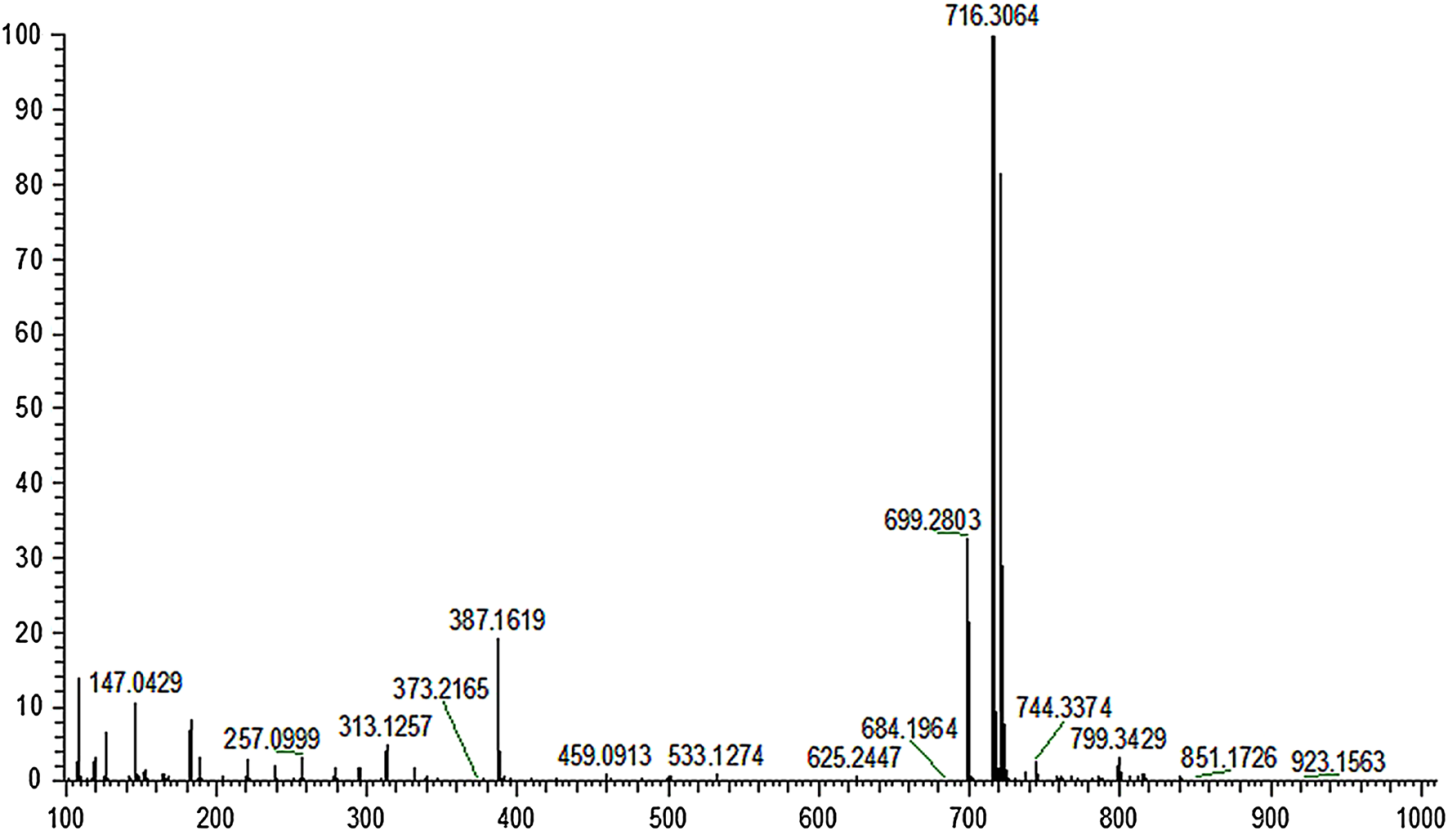
HRMS in PI mode at a retention time of 6.58 min.

The ionization pattern has a significant effect on the detection and characterization of analytes in the samples. In this study, positive ionization (PI) and negative ionization (NI) modes were tried: Fig. 1 shows TIC of CP-6 under the simulated gastric juice for 3 h at negative scanning mode (Upper section) and positive scanning mode (Lower section). Figure 2 is the HRMS in PI mode at a retention time of 6.58 min. M/z 699.2803 and m/z 716.3064 are the [M + H]^+^ peak and [M + NH_4_]^+^ peak of CP-6, respectively. It can be seen that there is no obvious peak of CP-6 at retention time ~ 6.5 min, and therefore the PI mode was selected in this study.

### Possible metabolic processes and accurate molecular weight of metabolic debris

**Figure 3:**
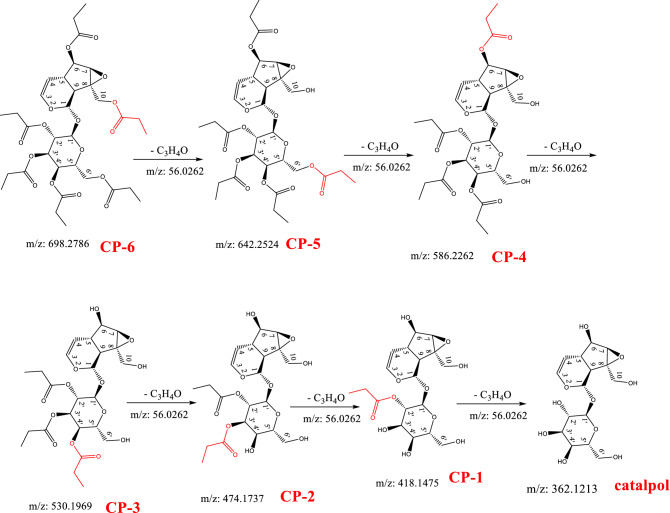
Possible metabolic process of CP-6 under simulated gastric conditions.

Based on the structure and synthesis procedure of CP-6, the possible metabolic process may be esterlysis of CP-6 that shown as Fig. 3.

In Fig. 3, the metabolites CP-5, CP-4, CP-3, CP-2, CP-1 and catalpol were produced by the hydrolysis of CP-6 and then the loss of 1 to 6 C_3_H_4_O–, i.e., loss of CH_3_CH_2_COO– and add an H–, respectively. The chemical formula and theoretical mass of ionized debris of the possible metabolites and CP-6 was shown in Table 1.

**Table 1:** Chemical formula and theoretical mass of ionized debris.

No.	Component (M)	Chemical Formula	m/z [M+H]^+^	m/z [M+NH_4_]^+^	m/z [M+Na]^+^
1	CP-6	C_33_H_46_O_16_	699.2864	716.3124	721.2678
2	CP-5	C_30_H_42_O_15_	643.2602	660.2862	665.2416
3	CP-4	C_27_H_38_O_14_	587.234	604.26	609.2154
4	CP-3	C_2__4_H_3__4_O_1__3_	531.2047	548.2307	553.1861
5	CP-2	C_2__1_H_3__0_O_1__2_	475.1815	492.2075	497.1629
6	CP-1	C_18_H_26_O_1__1_	419.1553	436.1813	441.1367
7	Catalpol	C_15_H_22_O_10_	363.1291	380.1551	385.1105

### Metabolites identification and semi-quantification

Since ESI is a typical “soft ionization” mode, generally does not occur decomposition during ionization, so we can identify the possible metabolites in mixtures by cross validation of their theoretical m/z of [M + H]^+^, [M + NH_4_]^+^, and [M + Na]^+^.

#### Metabolites identification

**Figure 4:**
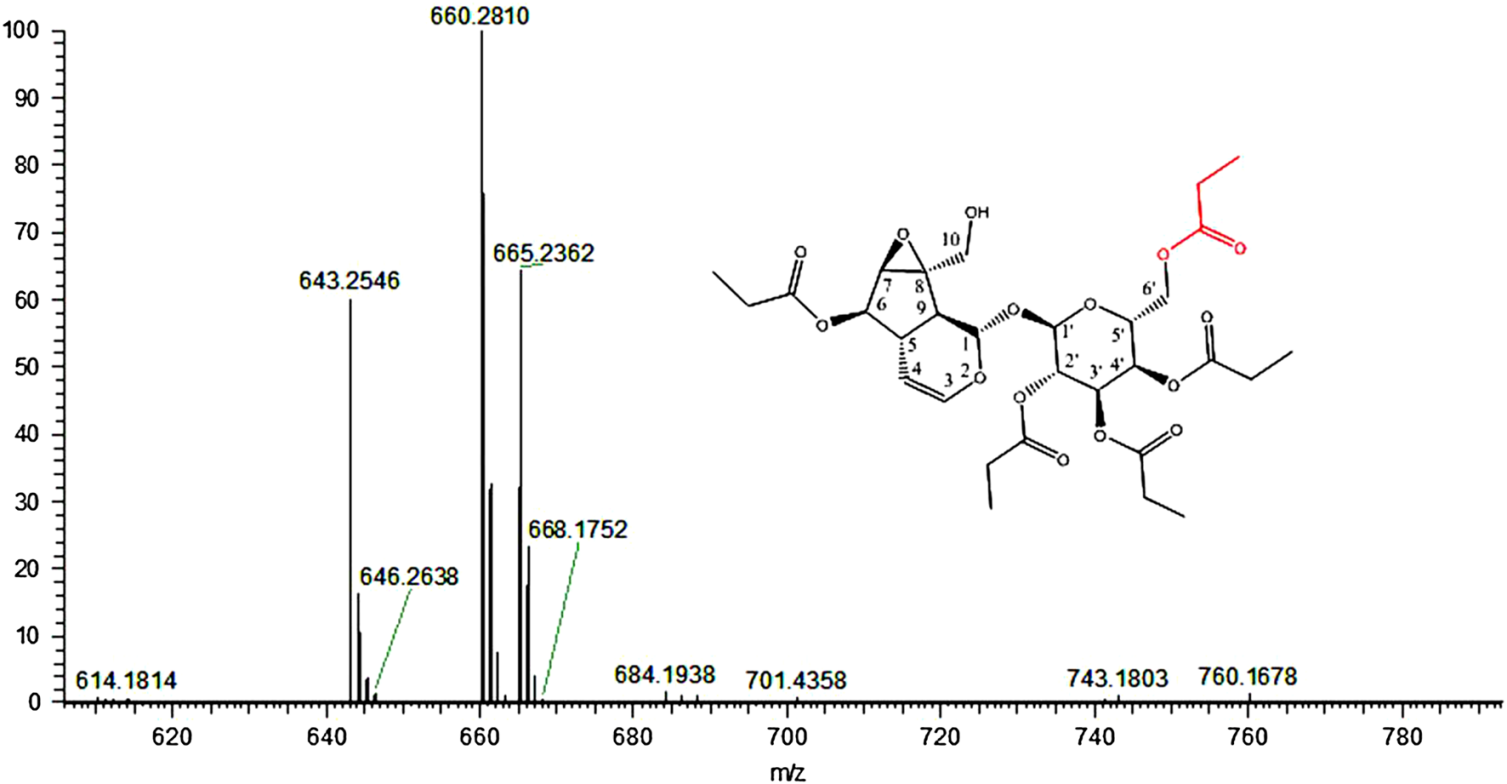
Experimental obtained HRMS in a mixed peak.

Take the identification of CP-5 as an example. Figure 4 shows HRMS of mixed peak at certain time. It can be seen that the experimental measured m/z 643.2546, 660.2810 and 665.2362 appeared that can be respectively to that the theoretical m/z [M + H]^+^, [M + NH_4_]^+^ and [M + Na]^+^ as 643.2602, 660.2862 and 665.2416 shown in Table 1, and the absolute deviations between the experimental and theoretical are less than 0.01. According to theoretical m/z of possible metabolites at PI mode, the experimental m/z of the metabolites were obtained and shown as Table 2. The identified metabolites are CP-5, CP-4, CP-3, CP-2, CP-1 and catalpol, which may be produced by the hydrolysis process of CP-6, or produced by esterificaion of the metabolites catalpol and propionic acid.

**Table 2:** Experimental m/z of metabolites at PI mode.

No.	Metabolite (M)	Chemical Formula	m/z [M+H]^+^	m/z [M+NH_4_]^+^	m/z [M+Na]^+^
1	CP-6	C_33_H_46_O_16_	699.2803	716.3065	721.2617
2	CP-5	C_30_H_42_O_15_	643.2546	660.2810	665.2362
3	CP-4	C_27_H_38_O_14_	587.2290	604.2556	609.2101
4	CP-3	C_2__4_H_3__4_O_1__3_	531.2046	548.2304	553.1846
5	CP-2	C_2__1_H_3__0_O_1__2_	475.1841	NA*	497.1583
6	CP-1	C_18_H_26_O_1__1_	419.1536	NA	441.1338
7	Catalpol	C_15_H_22_O_10_	NA	NA	385.0892

#### Metabolites quantification

**Figure 5:**
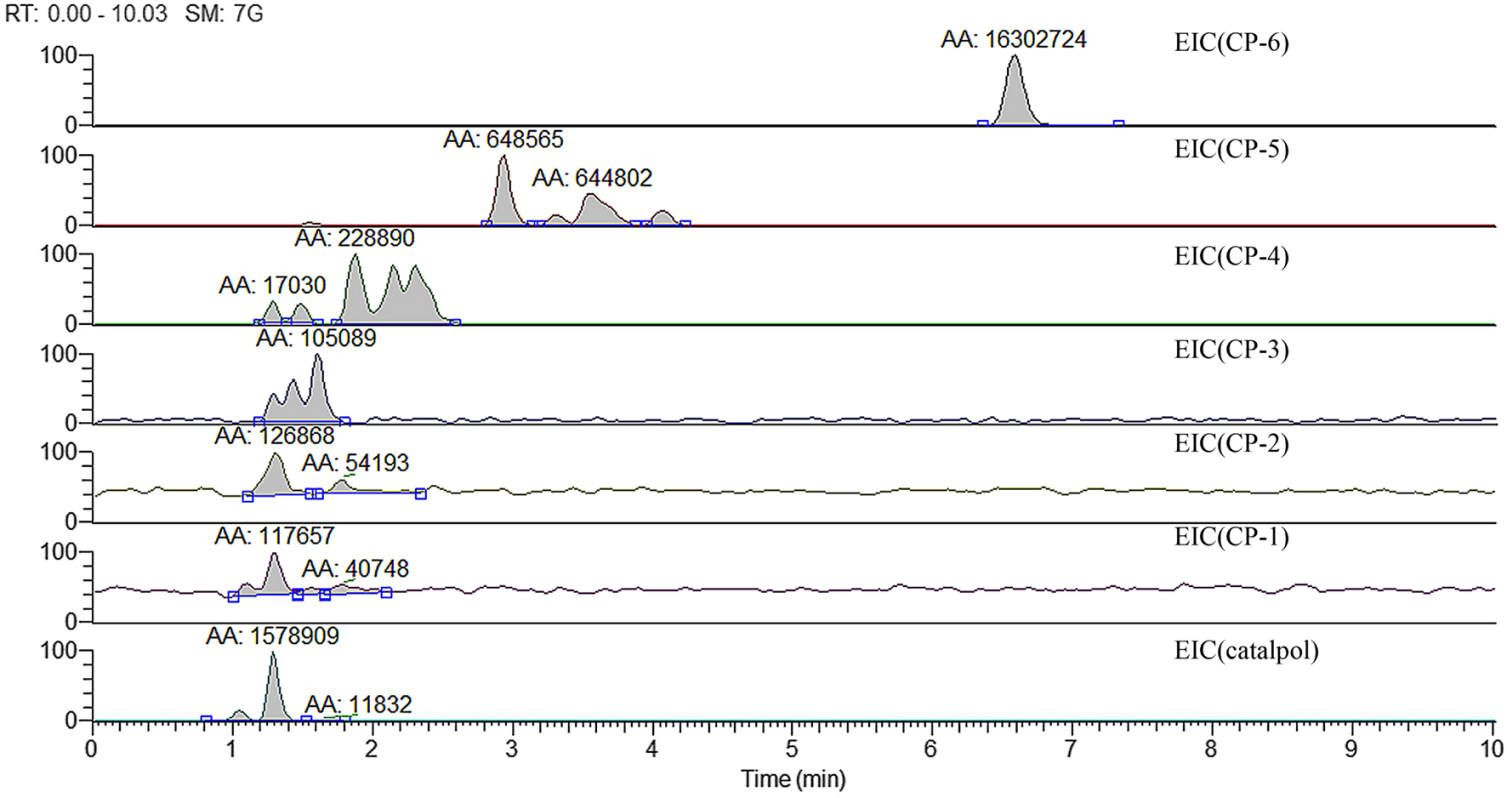
EIC of the metabolites at 3 h.

Because of the similar structures, the metabolites are difficult to be separated by UPLC. Although there five metabolites were identified, the TIC peaks analysis of the metabolites are are mainly at time (min) 1.09, 1.50 and 6.58. In order to quantify the metabolites, extracted ion chromatograms (EIC) of metabolites m/z [M + H]^+^, [M + NH_4_]^+^ and [M + Na]^+^ shown as Table 2 were used. The relative contents of the peak areas of the metabolites can represent their relative contents at certain time. Figure 5 shows EIC of the metabolites that CP-6 under the simulated gastric juice for 3 h. It should be noticed that the EIC of CP-5, CP-4, CP-3, CP-2 and CP-1 are with multiple peaks, and this is due to these components are multiple isomers^15^.

Table 3 shows the relative content changes of the metabolites within 4 h of CP-6 under the simulated gastric juice conditions using EIC mode based on the relative peak areas of the selected metabolites.

**Table 3:** Relative content changes of metabolites within 4 h.

Time/h	Relative content/%						
	CP-6	CP-5	CP-4	CP-3	CP-2	CP-1	Catalpol
0	99	0	0	0	0	0	0
0.05	91	4	0	0	0	0	5
0.5	84	7	1	0	1	0	6
1.0	83	6	2	0	0	1	8
1.5	88	5	1	0	0	0	5
2.0	83	6	1	0	1	0	9
2.5	82	6	1	0	0	0	10
3.0	81	7	1	1	1	1	8
3.5	82	6	1	0	0	0	10
4.0	84	6	1	1	1	1	7

It can be seen from Table 3 that CP-6 is unstable in the simulated gastric juice conditions, and the main metabolites are mainly CP-5 and catalpol; in first 0.5 h, CP-6 is rapidly hydrolyzed with the relative content decreased ~ 15%, while after 0.5 h it is generally stable; there are a spot of CP-4, CP-3, CP-2 and CP-1 produced, and this is may be due to the esterification of catalpol with the produced metabolite propionic acid^15^, or ester decomposition of C5, C4, C3 and C2. CP-6 and the metabolites are all have potential pharmacological effects which indicate that CP-6 is a prospective drug.

## Conclusions

The metabolites of CP-6 under simulated gastric conditions by UPLC-ESI-HRMS were mainly CP-5, catalpol and a spot of CP-4, CP-3, CP-2 and CP-1. The metabolic process of CP-6 may be an hydrolysis under acid conditions. It is rapidly decreased ~ 15% in the first 0.5 h and is generally stable after 0.5 h. The metabolites CP-5, CP-4, CP-3, CP-2, CP-1 and catalpol are all with pharmacological effects^15^. Therefore, although CP-6 is metabolized, it still has pharmacological activity. The research results indicate that CP-6 is a prospective drug. This study provides a theoretical basis for the development and utilization of CP-6 as a pharmaceutical preparation.

## Data Availability

The related data and materials are available from the corresponding author on reasonable request.
